# Preoperative Electrophysiology in Patients With Ulnar Nerve Entrapment at the Elbow-Prediction of Surgical Outcome and Influence of Age, Sex and Diabetes

**DOI:** 10.3389/fcdhc.2022.756022

**Published:** 2022-03-16

**Authors:** Ilka Anker, Erika Nyman, Malin Zimmerman, Ann-Marie Svensson, Gert S. Andersson, Lars B. Dahlin

**Affiliations:** ^1^ Department of Translational Medicine – Hand Surgery, Skåne University Hospital, Lund University, Malmö, Sweden; ^2^ Department of Hand Surgery, Skåne University Hospital, Malmö, Sweden; ^3^ Department of Biomedical and Clinical Sciences, Linköping University, Linköping, Sweden; ^4^ Department of Hand Surgery, Plastic Surgery and Burns, Linköping University Hospital, Linköping, Sweden; ^5^ National Diabetes Register, Centre of Registers, Gothenburg, Sweden; ^6^ Department of Molecular and Clinical Medicine, Institute of Medicine, University of Gothenburg, Gothenburg, Sweden; ^7^ Department of Neurophysiology, Skåne University Hospital, Lund University, Lund, Sweden

**Keywords:** diabetes, ulnar nerve, neuropathy, surgery outcome, axonal degeneration, QuickDASH

## Abstract

The impact of preoperative electrophysiology on outcome of surgical treatment in ulnar nerve entrapment at the elbow (UNE) is not clarified. Our aim was to evaluate influence of preoperative electrophysiologic grading on outcome and analyse how age, sex, and in particular diabetes affect such grading. Electrophysiologic protocols for 406 UNE cases, surgically treated at two hand surgery units reporting to the Swedish National Quality Register for Hand Surgery (HAKIR; 2010-2016), were retrospectively assessed, and graded as normal, reduced conduction velocity, conduction block or axonal degeneration. Outcome of surgery after primary and revision surgery was evaluated using QuickDASH and a doctor reported outcome measure (DROM) grading. No differences in QuickDASH or DROM were found between the four groups with different electrophysiologic grading preoperatively, or at three and 12 months or at follow up, respectively. When dichotomizing the electrophysiologic grading into normal and pathologic electrophysiology, cases with normal electrophysiology had worse QuickDASH than cases with pathologic electrophysiology preoperatively (p=0.046). Presence of a conduction block or axonal degeneration indicated a worse outcome by DROM grading (p=0.011). Primary surgeries had electrophysiologic more pronounced nerve pathology compared to revision surgeries (p=0.017). Cases of older age, men, and those with diabetes had more severe electrophysiologic nerve affection (p<0.0001). In the linear regression analysis, increasing age (unstandardized B=0.03, 95% CI 0.02-0.04; p<0.0001) and presence of diabetes (unstandardized B=0.60, 95% CI 0.25-0.95; p=0.001) were associated with a higher risk of a worse electrophysiologic classification. Female sex was associated with a better electrophysiologic grading (unstandardized B=-0.51, 95% CI -0.75- -0.27; p<0.0001). We conclude that older age, male sex, and concomitant diabetes are associated with more severe preoperative electrophysiologic nerve affection. Preoperative electrophysiologic grade of ulnar nerve affection may influence surgical outcome.

## Introduction

Ulnar nerve entrapment at the elbow (UNE) is mainly considered to be idiopathic. However, risk factors, such as age, sex, concomitant carpal tunnel syndrome (CTS), heavy manual work and multiple occasions of minor pressure at the retrocondylar groove, may predispose to the condition indicating surgery (1–4), but the factors also risks for UNE relapse requiring surgical revision (5–7). Furthermore, diabetes is a known risk factor for compression neuropathies, including UNE (3, 8–10).

The diagnosis of UNE is often based on patient history, symptoms, and clinical signs, supported by electrophysiologic findings (sensitivity 73-96%) to localize the site and estimating the severity of nerve compression (11–13). In addition, electrophysiologic examination may predict surgical outcome according to some studies (14, 15). However, outcome of primary simple decompression does not seem to differ between cases with solely clinical diagnosis compared to cases with a diagnosis supported by electrophysiology, indicating that clinical symptoms weigh heavily for diagnosis and treatment (14)*.* There is no clear consensus on optimal management of UNE, and the benefit of preoperative electrophysiology for diagnosis, and prognosis of surgery. There is also a debate about the impact of comorbidity, such as diabetes (16).

Outcome of surgical treatment for UNE seems to be similar (17–19) irrespective of surgical method, with respect to improvements in both clinical and electrophysiologic variables, and even regarding severity of UNE (16). Diabetes does not affect patient reported outcome after simple decompression in primary UNE, but men with diabetes have a risk for more residual postoperative symptoms (20). In addition, the relation between preoperative electrophysiologic grading and outcome in UNE patients with diabetes is not known.

There is a need for a clinically applicable preoperative electrophysiologic grading in UNE in order to predict outcome as related to patient characteristics as well as to comorbidities. Our aim was to evaluate the impact of the preoperative electrophysiologic grade of ulnar nerve pathology on outcome of surgery for UNE at the elbow with respect to age, sex, and diabetes.

## Methods

All surgically treated UNE cases between 2010-2016 [identified by ICD-10 diagnosis code G562 and surgical codes ACC53 (simple decompression), ACC43 (transposition) or NCK19 (medial epicondylectomy)], from two hand surgery departments (Malmö and Linköping) included in the Swedish National Quality Register for Hand Surgery (HAKIR; www.hakir.se) (21), were identified and cases with available preoperative electrophysiologic data were included in the study. Electrophysiologic protocols and medical charts were retrospectively assessed. The Swedish National Diabetes Registry (NDR; www.ndr.nu) for adults was merged with data from HAKIR to obtain data for patients with diabetes. The NDR contains data on type of diabetes treatment, complications, and associated risk factors (20, 22). Each case was defined as a treated nerve. The expression UNE was consistently used in the present study, independently exactly where the ulnar nerve was affected; thus, possibly at the level of the medial epicondyle or by the ligament of Osborne in accordance with previous publications (20, 23). The study was approved by the Regional Ethical Review Boards in Lund, Sweden (No 2016/931 and 2018/57) and Regional Ethics Review Board, Linköping, Sweden (register number 2016/88-31).

### Data From National Registries and Medical Charts

Data from HAKIR consisted of age, sex, type of ulnar nerve surgery, other concomitant hand surgical procedures, operated side and date of surgery. Pre- and postoperative disability were in the register assessed using the Swedish version of the patient reported outcome measure (PROM) QuickDASH (shortened version of the DASH; Disability of Arm, Shoulder and Hand questionnaire; total calculated score 0-100, higher score indicating more disability). Outcome was scored at three and 12 months postoperatively, as earlier described (20, 23–25).

Additional clinical data, not registered in HAKIR, was retrospectively sampled from patient charts as previously described (23). Doctor reported outcome measure (DROM) was based on the last out-patient visit (graded by IA; not treating surgeon in any case) and was graded into four groups; cured, improved, unchanged and worsened, and later dichotomized into two groups for statistical analyses (cured/improved and unchanged/worsened).

### Electrophysiology

Electrophysiologic examinations were performed on the ulnar nerves, in most cases bilaterally. The nerves were stimulated at the wrist, below and above elbow and a response was recorded from the abductor digiti minimi muscle. The patients in Lund, Sweden, were also examined with a short segment (2 cm) stimulation across the elbow segment. F-waves and orthodromic sensory response of the ulnar nerve to stimulation of the little finger were also recorded. The results were revised, assessed and graded by one of the authors (GS.A; specialist in neurophysiology; blinded to treatment and outcome) into four groups based on reference values at the Departments of Clinical Neurophysiology in Lund and Linköping, Sweden, respectively, with defined diagnostic criteria for the abnormal groups: i.e. (1) normal findings, (2) reduced conduction velocity across the elbow segment [if upper normal limits are exceeded for a single 2 cm segment (0.9 msec for men and 0.8 msec for women), two segments (1.3 msec men, 1.2 msec women) or all seven segments (3.3 msec men, 3.0 msec women)], (3) nerve conduction block (a 20% amplitude drop over the elbow segment when stimulating above elbow compared with stimulating below elbow), or (4) axonal degeneration [sensory and/or motor amplitudes below normal limit (dependent on age, sex and body height) as earlier described (14, 26). If a nerve showed both reduced conduction velocity and axonal degeneration, it was graded according to its most pathological parameter.

### Statistical Analyses

Data are presented as median [interquartile range; IQR; Q25-Q75]. Nominal data are presented as numbers (%). For nominal data, a Chi-squared test (Pearson or Fisher´s exact test) was used to compare differences between groups. Non-parametric Kruskal-Wallis test was used to compare differences between groups for continuous data, with subsequent *post-hoc* analyses (Mann-Whitney U test). Correlations were assessed by Point-Biserial correlation coefficient for dichotomous variables (r, with p-value). An r-value of ≥0.30 (positive or negative value) was interpreted as a correlation (0.30 – 0.7 = moderate correlation; >0.70 = strong correlation). Linear regression analyses were performed to analyse effects of nominal factors on QuickDASH score (unstandardized B [95% CI]; p-value). A linear regression analysis was performed to investigate the effect of another hand surgical procedure or surgery for another nerve entrapment performed at the same time as UNE surgery on QuickDASH results. All regressions were adjusted for age, sex and diabetes. A p-value <0.05 was considered statistically significant. IBM^®^ SPSS^®^ Statistics, version 26, 2019 (IBM Inc., Chicago, IL) was used for all calculations. Each treated arm was analysed as a separate case and statistical entity.

## Results

### Case Characteristics and Surgeries

**Figure 1:**
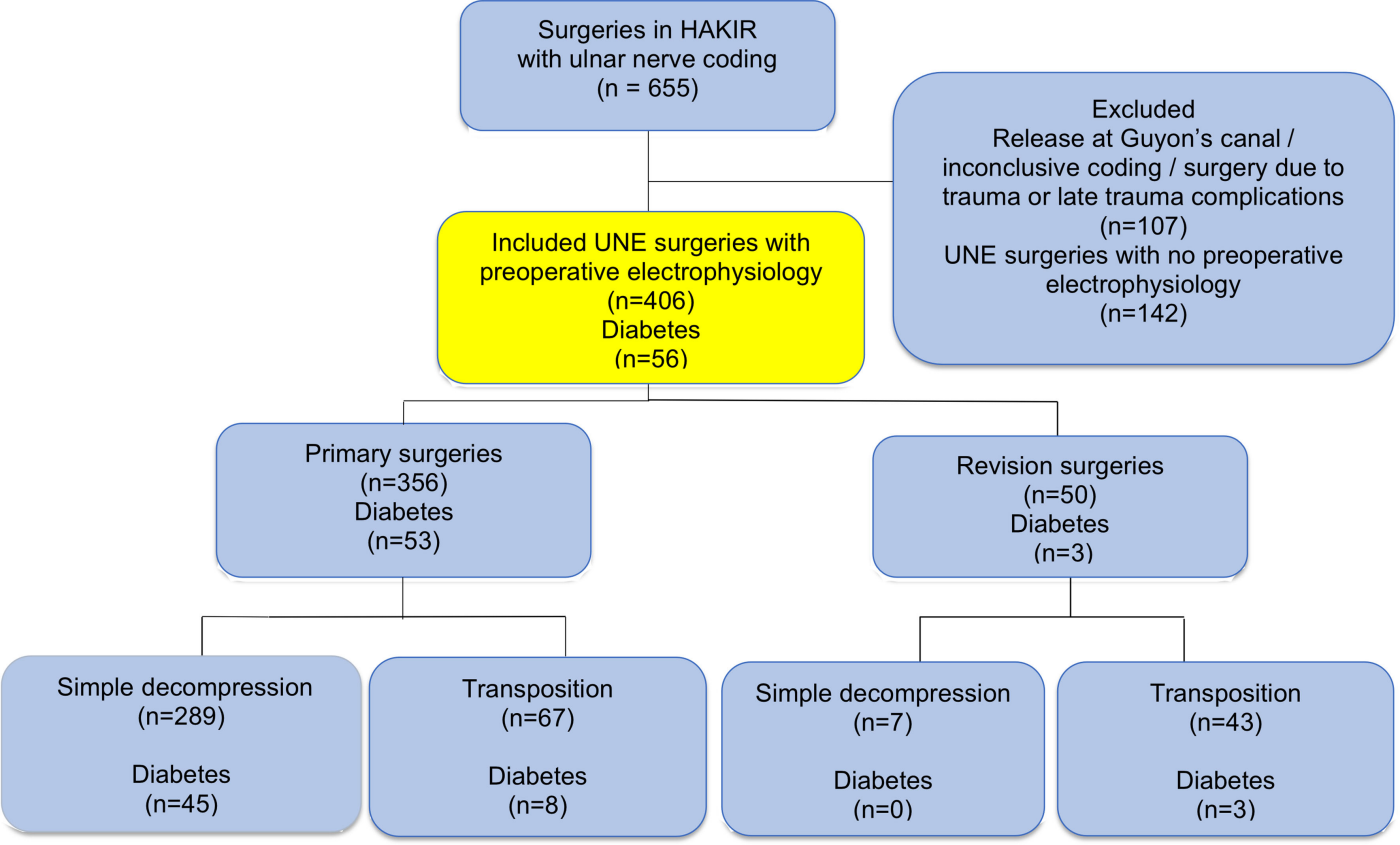
Flow chart describing the inclusion process of surgeries due to ulnar nerve compression from national quality register HAKIR (top blue), where preoperative electrophysiology examination was available of cases with ulnar nerve compression at the elbow (UNE) (yellow). Included cases are indicated and also specified concerning the number of cases with diabetes. For details see text.

A larger proportion of the patient cohort has been described earlier (23). Characteristics of the cases grouped by surgical procedure are presented in **Table 1**. Out of the original population, consisting of 548 UNE surgeries, solely 406/548 (74%) surgeries, on which preoperative electrophysiologic data was available, were included in the present study (**Figure 1**). Out of the included 406 UNE surgeries, 356/406 (88%) were primary surgeries (81% simple decompression) and 50/406 (12%) were reoperations (86% transpositions). There were no cases surgically treated with a medial epicondylectomy or endoscopic decompression (**Table 1**).

**Table 1:** Characteristics of 406 surgically treated cases with ulnar nerve entrapment (UNE) grouped by surgical procedure performed at two hand surgery units.

	Primary simple ulnar nerve decompression (n = 289)	Primary ulnar nerve transposition (n = 67)	Revision simple ulnar nerve decompression (n = 7)	Revision ulnar nerve transposition (n = 43)
Age, years	53 [45-62]	48 [38-60]	40 [32-54]	51 [41-57]
Male/female	148 (51%)/141 (49%)	31 (46%)/36 (54%)	4 (57%)/3 (43%)	16 (37%)/27 (63%)
Diabetes	45 (16%)	8 (12%)	0 (0%)	3 (7%)
Electrophysiology supporting UNE diagnosis	209 (72%)	39 (58%)	3 (43%)	21 (49%)
Concomitant hand surgical procedure(s)	15 (5%)	7 (10%)	0 (0%)	1 (2%)
Concomitant nerve surgical procedure(s)	33 (11%)	5 (8%)	1 (14%)	8 (19%)
				
Preoperative QuickDASH score	54 [39-67]	64 [55-80]	NA	61 [23-70]
	(n = 70)	(n = 15)		(n = 7)
3 months postoperative QuickDASH	30 [14-58]	53 [42-61]	NA	39 [18-58]
	(n = 78)	(n = 12)		(n = 17)
12 months postoperative QuickDASH	39 [16-58]	48 [33-66]	NA	43 [32-64]
	(n = 81)	(n = 9)		(n = 11)
				
DROM cured/improved	210 (73%)	53 (79%)	4 (57%)	28 (65%)
DROM unchanged/worsened	70 (24%)	13 (20%)	3 (43%)	14 (33%)
Missing DROM judgment	9 (3%)	1 (2%)	0 (0%)	1 (2%)

Data are presented as median [IQ range; Q25-Q75] or as numbers (%) for nominal data. NA; not applicable due to few cases, DROM; doctor related outcome measure (i.e. four-grade scale to evaluate outcome, dichotomized into cured/improved and unchanged/worsened) (14, 26).

Out of all surgically treated cases, 207/406 (51%) were females, with a median age of 50 [interquartile range; IQR 41-59] years for women and 53 [44-62] for men (**Table 1**). In total, 56/406 (14%) had concomitant diabetes [16/56 (29%) with type 1 and 35/56 (62%) with type 2 diabetes, data missing or unclassified in 5 cases; 9%]. Another hand surgical procedure was performed at the same time as the surgery for UNE in 6% of cases; i.e. surgery for trigger finger, thumb basal osteoarthritis, ganglion, de Quervain’s tenosynovitis or idiopathic synovitis. Another nerve entrapment surgery was performed at the same time as the surgery for UNE in 12%; i.e. carpal tunnel release, decompression of the ulnar nerve at wrist level (Guyon´s canal), decompression of the radial nerve or surgery on multiple nerves. These concomitant hand surgical and nerve related entrapment procedures (adjusted for age, sex, and diabetes) did not affect QuickDASH results at 3 or at 12 months (regression analysis; data not shown).

### Preoperative Electrophysiology and Surgical Procedures

Among primary surgeries, there were relatively more cases with reduced nerve conduction velocity and axonal degeneration, based on the electrophysiologic grading, than among revision surgeries (p=0.017; **Table 2**). Electrophysiologic grading preoperatively did not differ when comparing primary simple decompressions with primary ulnar nerve transpositions (p=0.07; results not shown). There were too few cases among revision simple decompression surgeries with electrophysiologic pathology [3/50 (6%)] for adequate statistical analyses to be made on revision surgeries (data not shown).

**Table 2:** Relation between electrophysiologic grading and type of surgery in 406 surgically treated cases with ulnar nerve entrapment.

Electrophysiologic grading	Primary surgery (n = 356)	Revision surgery (n = 50)	P-value
Normal (n = 134)	108 (30%)	26 (52%)	
Reduced nerve conduction velocity (n = 60)	56 (16%)^a^	4 (8%)	
Conduction block (n = 23)	22 (6%)	1 (2%)	
Axonal degeneration (n = 189)	170 (48%)^a^	19 (38%)	**0.017**

Data are presented as numbers (%). Statistical differences are detected using the Chi-square test (p-values comparing all groups) and with subsequent analysis. P-value in bold indicates statistical significant.

a Significantly more cases with reduced nerve conduction velocity and axonal degeneration in the primary surgery group.

### Responders, PROM, DROM and Electrophysiology

QuickDASH response rates were 92/406 (23%) preoperatively, 107/406 (27%) at three months postoperatively and 101/406 (25%) at 12 months postoperatively. DROM grading (median follow up time 3.0 months [IQR 1.5-6.0]), was possible to evaluate in 395/406 (97%; missing in 11 cases; 3%) of cases. In the remaining cases, no postoperative outcome was noted in the patient charts.

When analysing all surgically treated UNE cases, no difference in QuickDASH was found neither preoperatively, nor at three or 12 months postoperatively, between the four electrophysiology groups (**Table 3**). Similar results were found when analysing solely primary UNE surgeries, i.e. no significant differences in QuickDASH score in relation to electrophysiologic grading preoperatively or at three or 12 months postoperatively (p=0.14 preoperatively; p=0.79 at 3 months; p=0.07 at 12 months postoperatively; data not shown). QuickDASH response rates were too low among revision surgeries for statistical analyses to be made.

**Table 3:** Relation between electrophysiologic grading and postoperative outcome using QuickDASH or DROM grading score in 406 and 395, respectively, surgically treated cases with ulnar nerve entrapment, irrespective of surgical method.

QuickDASH	Normal (n = 134)	Reduced nerve conduction velocity (n = 60)	Conduction block (n = 23)	Axonal degeneration (n = 189)	P-values
Preoperatively	61 [43-73]	53 [27-62]	34 [16-60]	55 [39-68]	0.09
	(n = 27)	(n = 14)	(n = 4)	(n = 47)	
3 months postoperatively	39 [22-60]	35 [15-48]	16 [10-72]	31 [15-63]	0.51
	(n = 33)	(n = 20)	(n = 8)	(n = 48)	
12 months postoperatively	45 [25-64]	24 [13-36]	43 [15-60]	48 [24-63]	0.06
	(n = 27)	(n = 18)	(n = 13)	(n = 44)	
					
**DROM grading** ^a^	**Normal (n = 134)**	**Reduced nerve conduction velocity (n = 60)**	**Conduction Block (n = 23)**	**Axonal degeneration (n = 189)**	
Cured/improved (n = 295)	105 (78%)	48 (80%)	15 (65%)	127 (67%)	
Unchanged/worsened (n = 100)	27 (20%)	10 (17%)	6 (26%)	57 (30%)	0.08
Missing grading^a^	2 (2%)	2 (3%)	2 (9%)	5 (3%)	

Data are presented as median [IQ range; Q25-Q75] or numbers (%). Statistical differences are detected using the Kruskal-Wallis test or the Chi-square test (p-value refer to all DROM groups). DROM, doctor related outcome measure (four-grade scale to evaluate outcome, dichotomized into cured/improved and unchanged/worsened) (26).

a Missing cases with DROM judgment excluded in statistical analysis.

When analysing DROM grading in all surgically treated cases (p=0.08; **Table 3**) and solely primary UNE cases (p=0.07; data not shown), no differences were found in postoperative outcome in relation to the four electrophysiologic grades of nerve affection.

Furthermore, when dichotomizing the electrophysiologic grading into normal [n=132; cured/improved=105 (80%) and unchanged/worsened=27 (20%)] and pathologic [n=263; cured/improved=190 (72%) and unchanged/worsened=73 (28%)] electrophysiology, no difference was observed in the DROM grading (p=0.14; Fisher’s exact test). Using the same dichotomizing procedure, the QuickDASH scores differed preoperatively (normal 61 [43-73], n=27; pathologic 55 [34-64], n=65; p=0.046), but not at three (39 [22-60], n=33; 30 [14-57], n=76; respectively, p=0.16) and 12 months (45 [25-64], n=27; 41 [15-59], n=75, respectively; p=0.31).

When the electrophysiologic grading was divided into two other groups in accordance with a previous method (14), normal and reduced velocity [n=190; cured/improved=153 (81%) and unchanged/worsened=37 (19%)] versus conduction block and axonal degeneration [n=205; cured/improved=142 (69%) and unchanged/worsened=63 (31%)], a significant difference was observed in grading with DROM (p=0.011; Fisher’s exact test). Using the same procedure, the QuickDASH scores did not differ preoperatively (normal/reduced velocity 59 [40-71], n=41; conduction block/axonal degeneration 55 [39-68], n=51; p=0.38), at three (39 [19-58], n=53; 30 [14-63], n=56; respectively, p=0.50) or 12 months (34 [18-55], n=45; 45 [21-62], n=57, respectively; p=0.25).

No moderate or strong correlations were found between neither electrophysiologic grading and pre- or postoperative QuickDASH scores, nor DROM grading.

### Age, Sex, and Electrophysiology

Cases at older age (regardless of sex and both in all and solely primary UNE) had more severe electrophysiologic findings than cases at younger age (p<0.0001; **Table 4**). A moderate positive correlation (r=0.38, p<0.0001) was found between age and electrophysiologic grade of nerve affection.

**Table 4:** Relation between electrophysiologic grading and age of the patients, sex and diabetes (latter only primary cases) in 406 (all) and 356 (primary), respectively, surgically treated cases with ulnar nerve entrapment, irrespective of surgical method.

Age (years)	Normal (n = 134)	Reduced nerve conduction velocity (n = 60)	Conduction block (n = 23)	Axonal degeneration (n = 189)	P-values
	45 (37-52)^a^	53 (45-59)	60 (58-65)	56 (47-64)	**<0.0001**
**Sex**	**Normal (n = 134)**	**Reduced nerve conduction velocity (n = 60)**	**Conduction block (n = 23)**	**Axonal degeneration (n = 189)**	
Male (n = 199)	47 (24%)	26 (13%)	10 (5%)	116 (58%)^b^	
Female (n = 207)	87 (42%)	34 (16%)	13 (6%)	73 (35%)	**<0.0001**
**Diabetes status** (only primary surgeries included)	**Normal (n = 108)**	**Reduced nerve conduction velocity (n = 56)**	**Conduction block (n = 22)**	**Axonal degeneration (n = 170)**	
Diabetes (n = 53)	2 (4%)	11 (21%)	5 (9%)	35 (66%)	
No diabetes (n = 303)	106 (35%)^c^	45 (15%)	17 (6%)	135 (44%)	**<0.0001**

Data are presented as numbers (%) for nominal data or median [IQ range; Q25-Q75] for continuous data. Statistical differences are detected using the Chi-square test (Pearson or Fisher´s; p-values indicated comparing all groups and with subsequent analysis below) or the Kruskal-Wallis test with posthoc Mann-Whitney U-test. P-values in bold indicate statistical significant.

a Cases with normal electrophysiology were significantly younger than those in the other three groups. Those with reduced conduction velocity were younger than cases with conduction block; the latter also being older than those with axonal degeneration.

b Significantly more men with axonal degeneration.

c Significantly more cases without diabetes among those with normal electrophysiology.

Men more often had axonal degeneration at the electrophysiologic examination than women (p<0.0001; **Table 4**). No correlation (moderate or strong) was found between electrophysiologic grading and sex.

### Diabetes and Electrophysiology

Cases with diabetes who had undergone primary surgeries were older (58 [IQR 53-64] years; n=53) compared to cases without diabetes (51 [IQR 42-61]) (p<0.0001; n=303; Mann-Whitney U-test), but with no differences in sex distribution (p=0.13; Fisher´s exact test). In addition, there was no significant difference in sex distribution among all the cases concerning presence of diabetes (males with diabetes 32/199 (16%) and females with diabetes 24/207 (12%); p=0.12; Fisher´s exact test). Most cases with diabetes were found among primary surgeries (53/56; 95%). Among revision surgeries only three cases had concomitant diabetes [3/56 (5%); among revision with ulnar nerve transpositions only; **Table 1**] and due to the low frequency, further analyses on and including revision surgeries were not performed.

Primary UNE cases with diabetes (only primary cases analysed due to few revision cases among patients with diabetes) had significantly more severe electrophysiologic pathology, in the form of reduced nerve conduction velocity, nerve conduction block and axonal degeneration compared to cases without diabetes (p<0.0001; **Table 4**), which was also found to be similar for men with diabetes (p=0.012) and women with diabetes (p=0.011; **Table 5**). No moderate or strong correlations were found between concomitant diabetes and electrophysiologic grade of nerve pathology.

**Table 5:** Relation between electrophysiologic grading and diabetes among men and women in 356 surgically treated cases with primary ulnar nerve entrapment.

Diabetes status and sex	Normal (n = 38)	Reduced nerve conduction velocity (n = 25)	Conduction block (n = 10)	Axonal degeneration (n = 106)	P-values
Males with diabetes (n = 31)	0 (0%)	4 (13%)	2 (6%)	25 (81%)	
Males with no diabetes (n = 148)	38 (26%)^a^	21 (14%)	8 (5%)	81 (55%)	**0.012**
**Diabetes status and sex**	**Normal (n = 70)**	**Reduced nerve conduction velocity (n = 31)**	**Conduction block (n = 12)**	**Axonal degeneration (n = 64)**	
Females with diabetes (n = 22)	2 (9%)	7 (32%)	3 (14%)	10 (45%)	
Females with no diabetes (n = 155)	68 (44%)^b^	24 (15%)	9 (6%)	54 (35%)	**0.011**

Data are presented as numbers (%). Statistical differences are detected using the Chi-square test (Pearson or Fisher´s; p-values indicated comparing all groups and with subsequent analysis below). P-values in bold indicate statistical significant.

a Significantly more males without diabetes among those with normal electrophysiology.

b Significantly more females without diabetes among those with normal electrophysiology.

### Linear Regression, Age, Sex, and Diabetes

Increasing age (unstandardized B=0.03, 95% CI 0.02-0.04; p<0.0001) and concomitant diabetes (unstandardized B=0.60, 95% CI 0.25-0.95; p=0.001) were associated with a higher risk of a worse electrophysiology classification, while female sex was associated with better grading in the electrophysiology classification (unstandardized B=-0.51, 95% CI -0.75- -0.27; p<0.0001).

## Discussion

In the present study, we found no significant differences in outcome, evaluated with QuickDASH or DROM, in all surgically treated cases or in solely primary cases, neither at three nor at 12 months postoperatively or at follow-up, respectively, when four different grades of electrophysiologic pathology were compared. This is in line with a previous systematic review reporting effectiveness and safety of treatment for UNE referencing few studies with a follow-up longer than 12 months (16). However, in accordance with our previous retrospective study (14), dichotomizing patients with a preoperative nerve conduction block or axonal degeneration against normal findings and reduced conduction velocity, a higher risk of worse postoperative outcome after primary simple decompression was found when outcome was analysed with DROM (14), but not with QuickDASH, even though DROM and QuickDASH has been found to be related (27). No statistical correlation analysis between individual nerve conduction velocities (in m/s) and QuickDASH scores was performed due to the limited number of cases. Electrophysiologic grading is not always considered in larger systematic reviews and meta-analysis when evaluating safety and outcome of surgical procedures for UNE (28). However, it is still a debate if and how the preoperative electrophysiologic grading influence outcome of surgery, which may depend on the methods of evaluation (29, 30).

One cannot exclude that a relation exists between the different severities of electrophysiologic grading and outcome as evaluated by QuickDASH, although significance was not achieved among the four groups, which may be related to statistical power. An explanation of our findings, regarding outcome when QuickDASH was used, may be due to a limited number of cases in each group and being an effect of under-power. When a dichotomizing procedure was performed, dividing electrophysiologic grade into normal and pathologic findings, the former had a slightly higher Quick DASH score preoperatively (around 6 points), but with no differences at three or 12 months, indicating more disability preoperatively for cases with normal electrophysiologic grading. This is clinically a minor difference in disability, meaning that those findings should be interpreted with caution. Nevertheless, the DROM grading indicated that if the patients present with the two worst electrophysiology grades, there is a risk of worse outcome, irrespective of being a nerve conduction block or presence of axonal degeneration. However, a larger population is required to distinguish the outcome of surgery based on the electrophysiology grades nerve conduction block and axonal degeneration. Furthermore, we cannot explain the present observation that there was an initial improvement in QuickDASH at three months and a subsequent worsening at 12 months among the patients with preoperative electrophysiology findings of axonal degeneration. One may speculate that such an affected nerve, due to the lower number of functioning nerve fibres, may be more susceptible to further trauma, such as development of scar tissue around the nerve over time.

In the current study, we found more cases with electrophysiologic more severe nerve pathology among primary surgeries compared to revision surgeries. In primary UNE cases, simple decompression is usually the surgical gold standard treatment, regardless of electrophysiologic severity of nerve affection (16). If an ulnar nerve dislocation is found pre- or perioperatively, an ulnar nerve transposition is commonly performed instead as the primary procedure. Even after a simple decompression a greater mobility of the ulnar nerve can be expected with a risk for dislocation of the ulnar nerve; a statement that is supported by a recent study (23). A significantly higher presence of ulnar nerve dislocation was found among revision surgeries compared to primary surgeries, and at the same time significantly higher presence of ulnar nerve dislocation among primary transposition surgeries was observed compared to primary simple decompressions (23). Hence, we interpret that our current findings might be reflecting a presence of ulnar nerve dislocation among revision surgeries and primary ulnar nerve transpositions, being the reason for these cases presenting an electrophysiological normal or less severe nerve pathology, i.e. due to these unstable ulnar nerves having normal electrophysiologic findings.

Further, we found that men and cases at older age had more severe electrophysiologic impact on nerve function compared to women and cases at younger age. For the latter, we also found a moderate positive correlation, altogether indicating that increasing age may affect electrophysiologic findings negatively and increase severity of nerve pathology. Some earlier studies point out that older age and male sex, among others, are risk factors to develop UNE (1–4). Men in the present study showed a higher proportion of axonal degeneration based on the electrophysiology examination, which also may indicate an increased susceptibility to compression. It has been shown that men have lower intraepidermal nerve fibre density in biopsies from skin at wrist level compared to women (31). This can be interpreted as men having a more sensitive peripheral nervous system, with less reserve nerve fibre capacity, being more prone to be affected by compression, compared to women and might support our findings of men, and particularly those with diabetes, having electrophysiologic more severe impact on nerve function. Data from national registers also support the notion that men with diabetes may not have the same benefit as women with diabetes to improve by a simple decompression (20).

When analysing comorbidity in form of concomitant diabetes, we found that cases with diabetes were significantly older, but there were no differences in sex distribution. Cases with diabetes had more severe electrophysiologic nerve pathology. Diabetes may affect the peripheral nervous system and is a known risk factor for distal sensory polyneuropathy (32) and compression neuropathies, such as carpal tunnel syndrome (33). Several studies have found diabetes to be a risk factor for primary UNE as well (3, 8–10), although it has not consistently been found to increase risk of UNE relapse (5–7). Diabetes affects peripheral nerves by inducing intraneural structural changes (34). We interpret our findings, of cases with diabetes having more severe electrophysiologic nerve affection, as a reflection of this known peripheral nerve affection due to the mentioned structural changes in the nerves.

The interpretation of our combined results, with male sex and diabetes as comorbidity being related to electrophysiological more severe nerve affection, might be explained by men having a more sensitive peripheral nervous system with less reserve capacity when it comes to nerve fibre quantity (31) and the fact that men, as reported, seem to be affected by diabetic neuropathy to a greater extent and earlier compared to women (35, 36).

### Strengths and Limitations

The low response rate in QuickDASH scores is a limitation even if similar rates have been reported in earlier studies. The HAKIR register was at the time of data collection (2010-2016) also a rather new register with initial problems to include patients. Due to the coding system in HAKIR, we did not have data on which type of transposition that was performed and data on whether surgery was primary or revision was not appropriately specified in HAKIR. Hence, the latter data was added after the thorough retrospective evaluation made on each unique patient chart. A further weakness is that we could not in detail, based on the information from the patient charts, define the exact level of ulnar nerve affection; thus, being at or just proximal to the medial epicondyle or distally, exactly at the ligament of Osborne (37), although the latter location was probably the most common site. However, we defined the presently used expression UNE as a single entity, including both locations, in accordance with previous publications (20, 23). A strength is the use of data from the two national quality registers (HAKIR and NDR), combined with data from each unique patient chart together with a validated outcome measure (QuickDASH), which enables analyses of outcome concerning a nationwide population.

## Conclusions

We conclude that older age, male sex, and diabetes are associated with more severe preoperative electrophysiologic nerve affection, which may be interpreted as more susceptible peripheral nerves in men and in diabetes that should be taken into account when surgically treating UNE patients. Preoperative electrophysiologic assessment and severe grade of ulnar nerve affection may influence surgical outcome.

## Data Availability Statement

The datasets generated and/or analysed during the current study are not publicly available. Public access to data is restricted by the Swedish Authorities (Public Access to Information and Secrecy Act; https://www.government.se/information-material/2009/09/public-access-to-information-and-secrecy-act/), but data can be available for researchers after a special review that includes approval of the research project by both an Ethics Committee at the national level (etikprövningsmyndigheten.se) and the authorities’ data safety committees (such as “KVB-decision”).

## Ethics Statement

The study was approved by the Regional Ethical Review Boards in Lund, Sweden (No 2016/931 and 2018/57) and Regional Ethics Review Board, Linköping, Sweden (register number 2016/88-31). The patients/participants provided their written informed consent to participate in HAKIR and NDR.

## Author Contributions

IA, EN, MZ, and LD generated the hypothesis and outline of the project. All authors interpreted the data and critically reviewed the report. IA and EN collected the data from electrophysiologic examinations and patients’ charts. GA analysed and interpreted the electrophysiologic data. A-MS was responsible for collecting the data from the diabetes register. IA performed the initial analyses and drafted the first manuscript. LD performed the final statistical analyses. A-MS and GA contributed to hypothesis generation and to writing the manuscript. All authors fulfilled the criteria for authorship. All authors contributed to the article and approved the submitted version.

## Funding

This study was supported by grants from the Lund University, ALF [grant number 2018-Project 0104], Region Skåne (Funds from Skåne University Hospital Malmö-Lund), the Swedish Diabetes Foundation [grant number DIA2016-117 and DIA2020-492], the Swedish Research Council [grant number 2021-01942], Sydvästra Skånes Diabetesförening, Sweden, and ALF Grants [grant number LIO-823361], Region Östergötland, Sweden.

## Conflict of Interest

The authors declare that the research was conducted in the absence of any commercial or financial relationships that could be construed as a potential conflict of interest.

## Publisher’s Note

All claims expressed in this article are solely those of the authors and do not necessarily represent those of their affiliated organizations, or those of the publisher, the editors and the reviewers. Any product that may be evaluated in this article, or claim that may be made by its manufacturer, is not guaranteed or endorsed by the publisher.
